# Development of *in vitro* and *in vivo* neutralization assays for New World alphaviruses based on the VSV pseudovirus system

**DOI:** 10.1080/22221751.2025.2555721

**Published:** 2025-09-01

**Authors:** Yawen Liu, Chunlong Chen, Binfan Liao, Xiuzhu Geng, Weijin Huang, Jianhui Nie

**Affiliations:** a Division of HIV/AIDS and Sex-Transmitted Virus Vaccines, National Institutes for Food and Drug Control (NIFDC), State Key Laboratory of Drug Regulatory Science, NHC Key Laboratory of Research on Quality and Standardization of Biotech Products, NMPA Key Laboratory for Quality Research and Evaluation of Biological Products, Beijing, People's Republic of China; b The Sixth Laboratory, National Vaccine & Serum Institute (NVSI), Beijing, People's Republic of China; c School of Life Science and Bio-pharmaceutics, Shenyang Pharmaceutical University, Shenyang, People's Republic of China

**Keywords:** New World alphaviruses, Pseudovirus, animal model, DNA vaccine, bioluminescence imaging

## Abstract

The New World alphaviruses, including Eastern Equine Encephalitis Virus (EEEV), Western Equine Encephalitis Virus (WEEV), and Venezuelan Equine Encephalitis Virus (VEEV), are known to cause neurological diseases that pose a significant threat to public health concerns and bioterrorism preparedness challenges due to their potential for aerosol transmission. Currently, no FDA-approved vaccines or antiviral drugs are available for humans, although ongoing studies are exploring potential solutions. Most vaccine evaluation methods rely on live virus models, which require handling in biosafety level 3 (BSL-3) facilities. In this study, we constructed pseudoviruses for NW alphaviruses using the vesicular stomatitis virus (VSV) system by expressing the glycoproteins E3-E2-E1 on the surface of the VSV vector. *In vitro* cell infection experiments revealed that the pseudovirus titres of EEEV and VEEV were comparatively higher. Bioluminescence imaging in a mouse model was used to assess infection *in vivo*. When injected into the brain, this was the main site of infection for NW alphavirus-based pseudoviruses. When administered via the tail vein, the pseudovirus primarily infected the spleen, while intraperitoneally injected pseudoviruses mainly infected the intestines and thymus. Furthermore, we systematically evaluated the correlation between neutralizing antibody titres induced by DNA immunization and the protection against homologous virus strains. This study establishes a safe, convenient, and efficient system for evaluating the protective effects of vaccines against NW alphaviruses, which can be operated in a BSL-2 facility.

## Introduction

**Figure 1.**
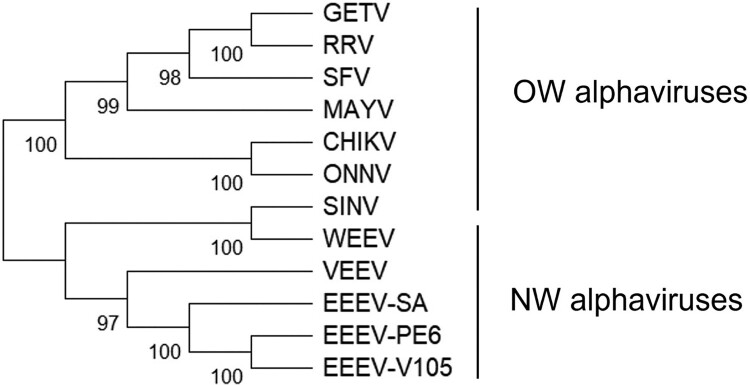
Unrooted phylogenetic tree of alphaviruses. Amino acid sequences of glycoproteins (E3, E2, E1) were retrieved from representative OW and NW alphaviruses. OW alphaviruses include Chikungunya (CHIKV), Getah (GETV), Mayaro (MAYV), O’nyong-nyong (ONNV), Ross River (RRV), Semliki Forest (SFV), and Sindbis (SINV). NW alphaviruses include EEEV V105, EEEV PE6, EEEV-SA BeAr436087, WEEV EQ237_2024, VEEV TC-83. The unrooted phylogenetic tree was constructed using the maximum likelihood (ML) method in MEGA11 and evaluated by bootstrap resampling with 500 replicates.

Alphaviruses, members of the Togaviridae family, are positive-sense single-stranded RNA viruses primarily transmitted by arthropod vectors [1]. The alphavirus genus comprises numerous species, categorized into Old World (OW) and New World (NW) alphaviruses. OW alphaviruses, such as Chikungunya virus (CHIKV), Mayaro virus (MAYV), Ross River virus (RRV), and O’nyong’nyong virus (ONNV), are generally less fatal but cause arthritogenic and febrile-rash diseases [2]. In contrast, NW alphaviruses, primarily found in the Americas, include Venezuelan equine encephalitis virus (VEEV), Eastern equine encephalitis virus (EEEV), and Western equine encephalitis virus (WEEV) (Figure 1). Infection with these viruses can lead to acute fever or severe neurological damage, with the potential for fatal outcomes in both humans and animals [3].

EEEV was first detected in horses in North America in 1831 and isolated in 1933 [4]. It was later recognized as a human pathogen, causing severe encephalitis [5]. Large-scale outbreaks occurred across northeastern and southeastern United States from the 1940s to 1960s, with sporadic cases emerging later [6]. EEEV infection causes severe neural damage, and studies have reported that the human mortality rates ranging from 30 to 70% [7]. There have been several reports indicating a gradual increase in the number of cases of EEEV in both animals (especially horses) and humans in the eastern seaboard of the United States as of December 2024. Its mortality rate is extremely high (close to 33%), making it a key focus of public health attention [8,9]. Based on antigenic specificity, there is another distinct subtype, South American (EEEV-SA), also known as Madariaga virus (MADV) [10]. The EEEV-SA strain had no clear association with human disease before 2010 [11,12]. So that there are limited in-depth epidemiological and ecological studies. In 2010, Darien et al. reported that MADV and VEEV were transmitted simultaneously in the population [13], but it is challenging to distinguish them from other arboviral diseases. VEE was first discovered in Venezuela in 1936 and was later recognized for its zoonotic mode of transmission [14,15]. From 1943 to 2003, outbreaks occurred successively in south american regions such as Colombia and Venezuela [16]. After an outbreak in Peru in 2006 [17], there have been few reports of related outbreaks. VEEV has a high mortality rate in horses (19–83%) [18,19], but the mortality rate in humans remains low (<1%), in spite of a high infection rate [20]. In 1930, WEEV was first isolated from horse cases in California, USA and was later recognized as a zoonotic virus [21]. After widespread outbreaks in the 1940s-1980s, WEEV cases had become rare [22], but it re-emerged in Argentina and Uruguay in 2023 [23,24]. WEEV typically causes mild or asymptomatic infections in humans [25], but progression to encephalitis has a much poorer prognosis, with a case fatality rate of 5–15% [26].

The alphavirus genome is approximately 11.5 kb and encodes four nonstructural and three structural proteins, including the E1, E2, and E3 glycoproteins, which are crucial for viral infectivity and virulence [27]. Although vaccines against these viruses have been developed for horses, no approved human vaccines exist. Currently, candidate vaccines for alphaviruses include live attenuated virus vaccines, inactivated virus vaccines, subunit vaccines, virus-like particles (VLP), chimeric vaccines, and nucleic acid vaccines [28–30].

The methods for evaluating vaccine efficacy include: (1) Immunological indicators (neutralizing antibody titres, detection of IgG/IgM antibodies, and cellular immune responses); (2) Virological indicators (plaque assay, real-time PCR); (3) Pathological indicators; and (4) Clinical indicators (such as body weight, temperature, survival rate, etc) [31]. Most aerosol infections with live viruses are conducted in BSL-3 facilities [32]. However, the pseudovirus system enables infection experiments, and vaccine protection evaluation experiments for high-risk viruses, to be conducted in a BSL-1/2 facilities both *in vitro* and *in vivo*.

E1 and E2 contain protective epitopes, serving as key antigenic determinants [33,34]. In this study, we established a VSV pseudovirus system for NW alphaviruses, expressing the glycoprotein E3-E2-E1 glycoprotein in place of the VSV envelope protein G. By using high-titre pseudoviruses, we can explore cell tropism *in vitro.* In mice, we established a dynamic and visual monitoring model for tissue distribution following infection. Additionally, we developed methods for evaluating the protective efficacy of DNA vaccines in animals. These approaches will accelerate the virological research progress on NW alphaviruses and improve the methods for evaluating vaccine efficacy.

## Results

### Construction of the NW alphavirus pseudoviruses based on the VSV system

To obtain NW alphavirus pseudoviruses, we selected the WEEV EQ237_2024 strain, VEEV TC-83 strain, and two EEEV-NA strains (V105 strain, PE6 strain) with different levels of virulence. To validate the functional differences and similarities between EEEV-SA and EEEV-NA strains in the pseudovirus system, we choose one EEEV-SA BeAr436087 strain. First, we analyzed the amino acid sequence similarity of the glycoproteins (E3, E2, E1) of NW alphaviruses. Multiple sequence alignment revealed 99.68% amino acid identity between EEEV-V105 and EEEV-PE6 glycoproteins, while the similarity between EEEV-V105 and EEEV-SA was 95.35%. However, the amino acid sequence similarity between EEEV, WEEV or VEEV ranged from 60% to 70%. The evolutionary relationships between the NW and representative OW alphaviruses are shown in Figure 1. Since SINV and WEEV both belong to the WEE serocomplex and share the common receptor avian MXRA8 [35], WEEV is more closely related to SINV.

**Figure 2.**
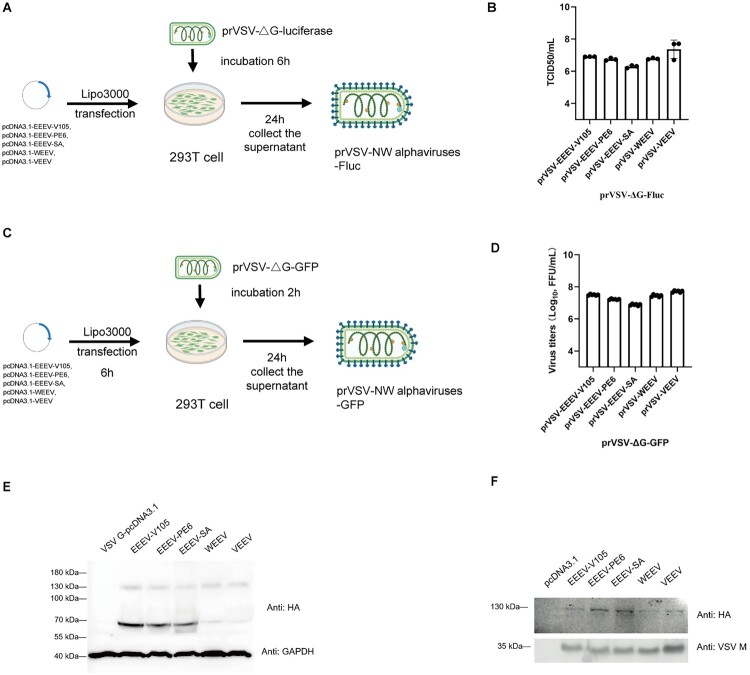
Construction of NW alphavirus pseudoviruses. (A) Schematic diagram of pseudovirus packaging. Vectors carrying the glycoprotein genes E3E2E1 of EEEV, WEEV, and VEEV were respectively used to transfect 293 T cells. Subsequently, pseudoviruses were packaged using prVSV-ΔG-luciferase. (B) The same amount of plasmid DNA (weight basis) was used for transfection in order to package pseudoviruses, which were then titrated using 4 × 10^4^ BHK21 cells/well. Three independent replicate experiments were performed. (C) Schematic diagram of the packaging of pseudoviruses encoding the GFP reporter gene. (D) The same amount of plasmid DNA (weight basis) was used for transfection in order to package pseudoviruses, which were then titrated using 4 × 10^4^ BHK21 cells/well. Six independent replicate experiments were performed. GFP positive cell numbers were counted 24 h post-infection using a fluorescent spot counter. Viral titre was expressed as focus-forming units per mL (FFU/mL). (E) Western blotting for glycoproteins of NW alphaviruses in 293 T cell. An anti-HA antibody was used to detect HA-E3E2E1 protein expression and an anti-GAPDH antibody served as the internal control. (F) Western blotting for NW alphavirus pseudoviruses. A HA antibody was used to detect the expression of glycoproteins of the pseudoviruses, with VSV M protein as the internal control.

Previous studies have reported a strong correlation in neutralization experiments using prVSV-SARS-CoV-2-Sdel21-GFP and prVSV-SARS-CoV-2-Sdel21-Fluc pseudoviruses [36]. Therefore, we constructed NW alphavirus pseudoviruses based on the VSV system with two reporter genes (GFP and Fluc) (Figure 2A, 2C), which were used for *in vitro* and *in vivo* experiments, respectively. These five plasmids (pcDNA3.1-EEEV-V105, pcDNA3.1-EEEV-PE6, pcDNA3.1-EEEV-SA, pcDNA3.1-WEEV, and pcDNA3.1-VEEV) were used to transfect 293T cells, and pseudoviruses were packaged using replication-deficient prVSV-ΔG-luciferase or prVSV-ΔG-GFP pseudoviruses. As a result, we successfully obtained high-titre NW alphavirus pseudoviruses (Figure 2B, 2D).

### Validation of glycoproteins expression in NW alphavirus pseudoviruses

Although implicated in glycoprotein processing and in virion assembly and release, the precise role of the 6 K/TF membrane protein was less well understood [37]. In this study, NW alphavirus pseudoviruses that did not include 6K/TF still exhibited high viral titres. Western blotting was used to detect the expression of NW alphavirus E3-E2-E1 proteins. The size of EEEV-V105-E3E2E1 was 924aa (E3 is 63aa, E2 is 420aa, E1 is 441aa), EEEV-PE6-E3E2E1 was 924aa (E3 is 63aa, E2 is 420aa, E1 is 441aa), EEEV-SA-E3E2E1 was 924aa (E3 is 63aa, E2 is 420aa, E1 is 441aa), WEEV-E3E2E1 was 922aa (E3 is 60aa, E2 is 423aa, E1 is 439aa), and that of VEEV-E3E2E1 was 924aa (E3 is 59aa, E2 is 423aa, E1 is 442aa). In addition, there is a conserved furin cleavage site at the C-terminus of E3 protein.

Since the glycoproteins were *N*-terminally tagged with the HA epitope, cell lysates were detected using the HA antibody, which revealed two target bands with approximate sizes of 130 and 70 kDa. These are currently hypothesized to be the full-length E3E2E1 protein (110 kDa) and the E3E2 precursor (55 kDa), respectively, both of which have undergone post-translational modifications[38,39] (Figure 2E). The weaker bands observed for WEEV and VEEV were likely attributable to lower levels of the E3E2 precursor (approximately 70 kDa), whereas the expression of the full-length HA-tagged protein in transfected cells appears comparable to that of EEEV. Numerous studies have investigated the functions of *N*-linked glycosylation in alphaviruses. The *N*-linked glycosylation of glycoproteins E2 and E1 promotes viral particle production, increases structural as well as functional diversity, and is conserved across alphaviruses [40]. After concentrating and ultracentrifuging the NW alphavirus pseudoviruses, the purified viral particles were analyzed by western blotting, with the M protein of VSV used as an internal control, as shown in Figure 2F. The results confirmed that the 130 kDa band corresponds to the full-length protein. The above results indicated that the pseudovirus particles were successfully packaged.

### Identification of susceptible cell lines for NW alphavirus pseudoviruses

**Figure 3.**
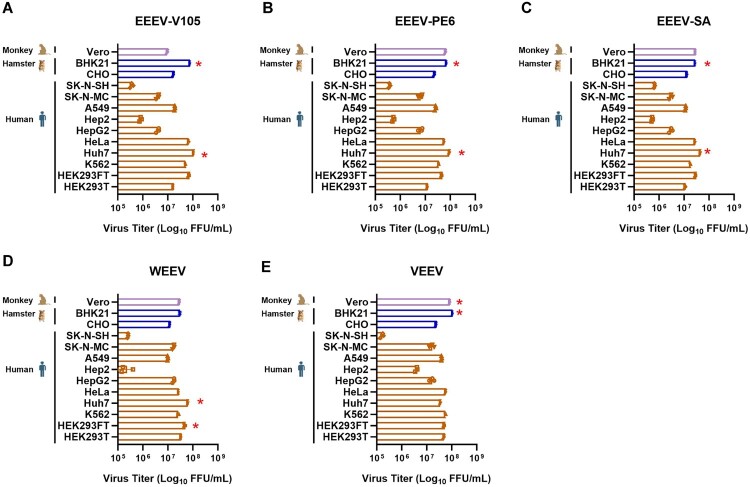
Detection of susceptible cell lines. After packaging NW alphavirus pseudoviruses to infect 293 T cells, pseudovirus titres were adjusted between 1.1 × 10^7^ and 5.8 × 10^7^ FFU/mL before proceed with the cell tropism experiment. Human-derived cell lines (293 T, 293FT, K562, Huh7, HeLa, HepG2, Hep2, A549, SK-N-MC, SK-N-SH), hamster-derived cell lines (CHO, BHK21) and a monkey-derived cell line (Vero) were adjusted to a density of 4 × 10^4^ cells/well. These cell lines were then infected with prVSV-EEEV-V105-GFP, prVSV-EEEV-SA-GFP, prVSV-EEEV-PE6-GFP, prVSV-WEEV-GFP, and prVSV-VEEV-GFP pseudoviruses. GFP positive cell numbers were counted 24 h post-infection using a fluorescent spot counter. Viral titre was expressed as focus-forming units per mL (FFU/mL).

Due to the different receptors used by NW alphaviruses, their infectivity and tropism also vary. This study utilized a pseudovirus system to investigate the tropisms of NW alphaviruses at the cellular level. EEEV-V105, EEEV-SA, EEEV-PE6, WEEV, and VEEV pseudoviruses were used to infect human-derived cell lines (293 T, 293FT, K562, Huh7, HeLa, HepG2, Hep2, A549, SK-N-SH, and SK-N-MC), a monkey-derived cell line (Vero), and hamster-derived cell lines (CHO, BHK21). The cellular tropism of these five NW alphavirus pseudoviruses showed significant differences (Figure 3). For EEEV, higher infectivity was observed in Huh7 and BHK21 cells (Figure 3A-C), which is consistent with previous studies where BHK21 cells have been used to detect the infectivity of alphaviruses [41]. The prVSV-WEEV pseudovirus showed higher infectivity in Huh7 and 293FT cells (Figure 3D), while prVSV-VEEV showed higher infectivity in BHK21 cells (Figure 3E). Vero cells were commonly used for the propagation of live alphaviruses [42]. This study also demonstrated that NW alphavirus pseudoviruses could efficiently infect Vero cells. Additionally, we acknowledge that these cell lines may not fully reflect the *in vivo* tissue tropism of NW alphaviruses. For example, NW alphaviruses have neurotropism, but the infection efficiency in neuroblastoma cell lines (SK-N-SH, SK-N-MC) was not the highest in this pseudovirus system, indicating that *in vitro* cell infection does not always correlate with *in vivo* tissue tropism in animals. The transcription and translation efficiency of the VSV genome may also influence the infectivity of the pseudovirus. The above results indicate that 293T cells were suitable for packaging pseudoviruses, while BHK21 cells were suitable for the infection experiments.

### Establishment of a mouse infection model using NW alphavirus pseudoviruses

**Figure 4.**
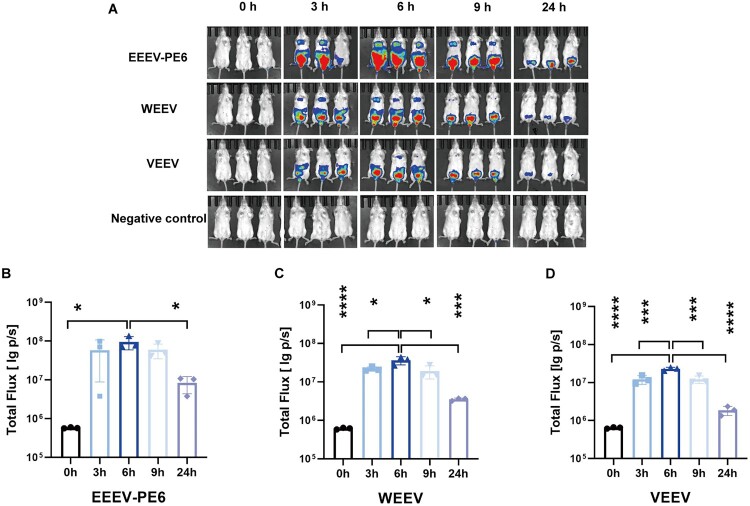
A dynamic and visual monitoring model of NW alphavirus pseudoviruses infection in mice. (A) The EEEV-PE6, WEEV, and VEEV pseudoviruses infection status of 6-week-old, female BALB/c mice at 0, 3, 6, 9, and 24 h after intraperitoneal (ip) injection of pseudoviruses. The negative control group was injected with the prVSV-pcDNA3.1 pseudovirus. In the negative control group, the same volume of the pseudovirus supernatant was administered as in the experimental group. (B-D) Total flux signals (p/s) for each mouse (3 mice in total) following inoculation with 3.6 × 10^5^ TCID50. Results were presented as means and standard deviations (SD). The statistical significance was assessed by one-way ANOVA followed by Tukey's post-hoc test within each group (*P < 0.05, **P < 0.01, ***P < 0.001, and ****P < 0.0001).

To establish a suitable animal infection model, we selected 6-week-old, female BALB/c mice. To explore the optimal time for biological imaging, we first administered the pseudovirus via intraperitoneal injection and conducted the bioluminescence imaging (BLI) values at 0, 3, 6, 9, and 24 h. For the EEEV-PE6 group, the one-way ANOVA results indicated that there were significant differences compared to the other groups (*F* = 5.41, *P*<0.05). The Tukey's multiple comparisons test showed that the values at 6 h were significantly higher than those at 0 h (*P*<0.05) and 24 h (*P*<0.05, Figure 4A, 4B). For the WEEV group, the one-way ANOVA results indicated that there were significant differences compared to the other groups (*F* = 23.54, *P*<0.0001). Tukey's multiple comparisons test showed that the values at 6 h were significantly higher than those at 0 h (*P*<0.0001), 3 h (*P*<0.05), 9 h (*P*<0.05), and 24 h (*P*<0.0001), (Figure 4A, 4C). For the VEEV group, the one-way ANOVA results indicated that there were significant differences compared to the other groups (F = 54.55, *P*<0.0001). The Tukey's multiple comparisons test showed that the values at 6 h were significantly higher than those at 0 h (*P*<0.0001), 3 h (*P*<0.001), 9 h (*P*<0.001), and 24 h (*P*<0.0001) (Figure 4A, 4D). These results indicated that 6 h was the optimal time for detection.

**Figure 5.**
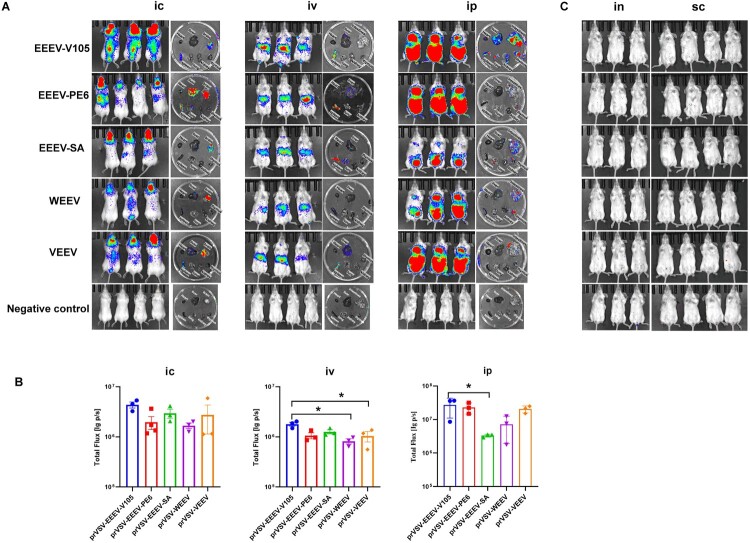
Establishment of the mice model using NW alphavirus pseudoviruses. The mice were infected through intracranial (ic), intravenous (iv), intraperitoneal (ip), intranasal (in), and subcutaneous routes (sc). (A) NW alphavirus pseudoviruses were administered to infect 6-week-old female, BALB/c mice via the ic (1.6×10^5^ TCID50/mouse), iv (3.2 × 10^5^ TCID50/mouse), and ip route (3.2 × 10^5^ TCID50/mouse). After 6 h, bioluminescence imaging (BLI) of the infected mice and dissected tissues were conducted. The negative control group was injected with the prVSV-pcDNA3.1 pseudovirus. (B) Values of total flux (lg) for the ic, iv, and ip groups. The statistical significance was assessed by one-way ANOVA followed by Tukey's post-hoc test within each group (*P < 0.05). The differences that are not significant are not marked. (C) NW alphavirus pseudoviruses were used to infect 6-week-old female BALB/c mice, three to four BALB/c mice per group, via the in (1.6 × 10^5^ TCID50/mouse) and sc (3.2 × 10^5^ TCID50/mouse) routes.

The pseudovirus was adjusted to a concentration of 3.2 × 10^6^ TCID50/mL and administered to mice via intraperitoneal, intravenous, intracranial, intranasal, and subcutaneous routes. The results showed that NW alphavirus pseudoviruses could not infect mice via subcutaneous or intranasal injection (Figure 5C). However, they were able to infect mice through intraperitoneal, intravenous, and intracranial routes (Figure 5A). When intracranial was administered, there were no significant differences in total bioluminescence signals (lg) among the groups, and high flux levels of dissected tissues were observed in the brain. Additionally, EEEV-V105, EEEV-PE6, and VEEV were detected in the spleen, with EEEV-PE6 showing significant infection in the liver.

Following intravenous infection, the bioluminescence signals of mice infected with EEEV-V105 were significantly higher than those of the VEEV and WEEV, and high levels of flux of dissected tissues were observed in the spleen. After infection with EEEV-PE6, signals were also detected in the liver. After infection with EEEV-SA, signals were also found in the lungs. Similarly, after infection with VEEV, signals were distributed in the liver (Figure 5A).

Following intraperitoneal challenge, the bioluminescence signals of mices infected with EEEV-V105 were significantly higher than of those infected with EEEV-SA, and high levels of flux of dissected tissues were observed in the intestines and thymus. After infection with EEEV-V105, signals were also detected in the liver, spleen, lungs, and kidneys. Additionally, a weak amount of signal was observed in the lungs after infection with EEEV-SA (Figure 5A). Therefore, ip infection with NW alphavirus pseudoviruses offered a more stable signal, while also being easy to perform.

### NW alphavirus DNA vaccine administration and detection of neutralizing antibody levels

**Figure 6.**
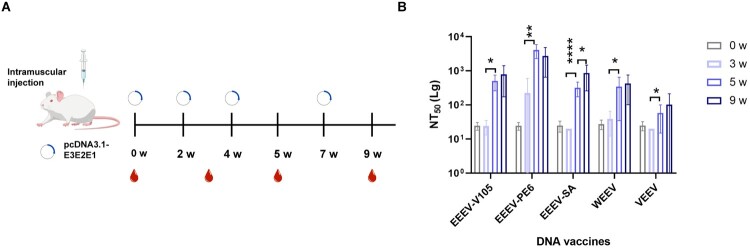
Plasmid DNA vaccine administration to elicit neutralizing antibodies (NAb) against NW alphaviruses. (A) Immunization schedule, 6 w, female BALB/c mice were immunized four times with 100 µg of plasmids DNA by intramuscularly injection. Blood samples were collected at 0 w, 3 w, 5 w, and 9 w. (B) NAb titres were detected at 0 w, 3 w, 5 w, and 9 w. Before each booster immunization, NAb titres were compared with those from the previous immunization using Student's *t*-test (*P < 0.05, **P < 0.01, ***P < 0.001, ****P < 0.0001).

The NW alphavirus DNA vaccine is composed of the following plasmids: pcDNA3.1-EEEV-V105, pcDNA3.1-EEEV-PE6, pcDNA3.1-EEEV-SA, pcDNA3.1-WEEV, and pcDNA3.1-VEEV (Figure 6A). After mice were immunized twice with 100μg of plasmids DNA, NAb levels were generally low. After three immunizations, NAb levels significantly increased. However, The NAb levels after the four immunization showed no significant difference compared with those after three immunizations for EEEV-V105, EEEV-PE6, WEEV, and VEEV (see the NAb levels detected in the blood samples collected at 9 w and 5 w). However, in the EEEV-SA group, NAb levels significantly increased after the fourth immunization (*P < 0.05) (Figure 6B). These data suggest that DNA vaccines for NW alphaviruses are capable of inducing high NAb levels.

**Figure 7.**
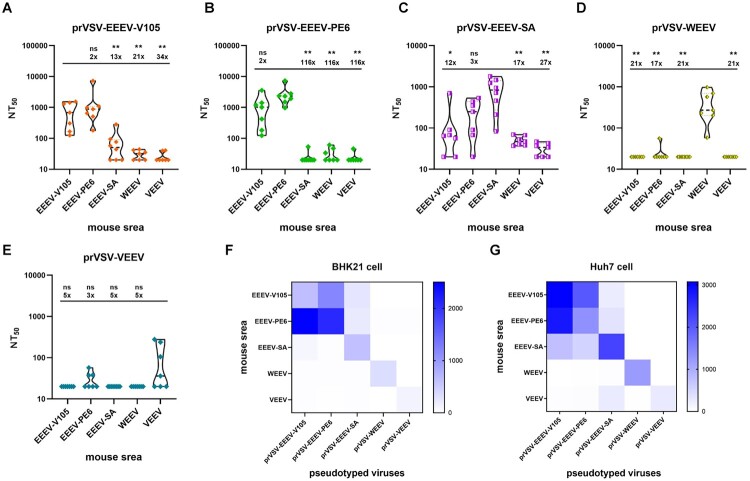
Comparison of NAb levels against the homologous viral strain and heterologous viral strains using NW alphavirus pseudoviruses. The prVSV-EEEV-V105-GFP, prVSV-EEEV-SA-GFP, prVSV-EEEV-PE6-GFP, prVSV-WEEV-GFP, and prVSV-VEEV-GFP pseudoviruses were neutralized with mouse sera elicited by plasmid DNA vaccination,with two duplicate wells per group. The NAb levels (NT_50_) were calculated after 24 h. (A-E) The neutralization assay was based on BHK21 cells. The data were analyzed using the Student's *t*-test. (F) Heatmap of Z-score normalized NAb levels based on an assay in BHK21 cells. (G) Heatmap of Z-score normalized NAb levels based on an assay in Huh7 cells.

To investigate the protective efficacy of DNA vaccines against NW alphaviruses for both homologous and heterologous strains, the first step was to examine the changes in NAb levels *in vitro*. The results showed that among EEEV, WEEV, and VEEV, there was no significant coss-protection against heterologous virus strains. However, a clear protective effect was observed against the parental virus strains (Figure 7). Immunization with EEEV-105 showed significant neutralizing activity against the EEEV-V105 and EEEV-PE6 strains, but only weak neutralizing activity against the EEEV-SA strain. Similarly, immunization with EEEV-PE6 elicited high NAb titres against the heterologous EEEV-V105 strain, but immunization with EEEV-SA showed weak neutralizing activity against the heterologous EEEV-V105 strain. Accordingly, the above results were validated in both hamster-derived BHK21 cells and human-derived Huh7 cells. In summary, immunization with the DNA vaccines against NW alphaviruses induced significant NAb against the homologous strains.

**Figure 8.**
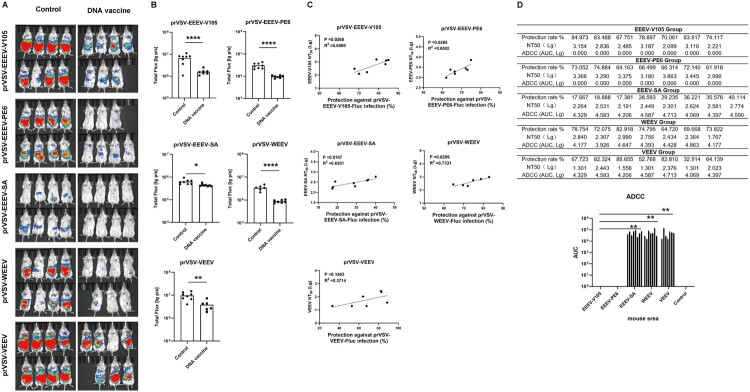
Protective immunization experiment. (A). Imaging of mice 6 h after NW alphavirus pseudovirus injection (3.2 × 10^5^ TCID50/mouse), *n* =  7–8 per group. (B) Analysis of total bioluminescence (C) Correlation analysis between vaccine-induced NAb levels and protective efficacy. (D) The table presented the protection rate (%), NAb levels (NT_50_), and ADCC activity (AUC) for individual mice in the EEEV-V105, EEEV-PE6, EEEV-SA, WEEV, and VEEV immunization groups. The graph below summarized the ADCC activity across these five groups. Data were analyzed using the Student's *t*-test (*P < 0.05, **P < 0.01, ***P < 0.001, and ****P < 0.0001).

DNA vaccines targeting NW alphaviruses elicited NAb against the homologous virus strains in mice and conferred protection against intraperitoneal challenge with the homologous virus strains. As shown in Figure 8, total flux in the DNA-immunized mice was significantly reduced after injection with EEEV-V105 pseudovirus compared to the control group (*P*<0.001), indicating that vaccination conferred 78% protective efficacy against challenge with the homologous strain challenge. More importantly, a significant moderate positive linear correlation was found between NAb levels and protective efficacy (R^2^ = 0.6580). For the EEEV-SA pseudovirus, the protection rate was only 28%, but this can be explained by the low infection efficiency. In addition, there was a strong linear correlation between NAb levels and protective efficacy after vaccination (R^2^ = 0.6301).

The total bioluminescence signals after injection with EEEV-PE6 pseudovirus were significantly reduced in the DNA-immunized mice compared with the control mice (*P*<0.001), indicating that DNA immunization provided approximately 68% protection against the EEEV-PE6 strain. Moreover, a moderate positive linear correlation between NAb levels and protection was observed (R^2^ = 0.6502).

Compared to control mice, the total bioluminescence value after injection with the homologous pseudovirus was significantly reduced in the mice immunized with VEEV (*P*<0.005), indicating that DNA immunization provided approximately 67% protection against the VEEV strain. However, only a weak positive correlation was observed between NAb levels in immunized mice and the protective efficacy (R^2^ = 0.3714).

For the WEEV DNA-immunized group, the average protection rate was 74%, and this study demonstrated a strong correlation between NAb levels and protection, which was statistically significant (R^2^ = 0.7331).

Based on these findings, we conducted ADCC assays to determine whether Fc-mediated effector functions contributed to the observed protective effects. In this study, we assessed the ADCC activity in mouse sera from the EEEV-V105, EEEV-PE6, EEEV-SA, WEEV, and VEEV immunization groups. The individual protection rates, NAb titres, and ADCC activities for each mouse in the EEEV-V105, EEEV-PE6, EEEV-SA, WEEV, and VEEV immunization groups were summarized in Figure 8D. The results showed that, compared with the EEEV-V105 or EEEV-PE6 groups, the EEEV-SA, WEEV, and VEEV groups elicited significantly higher levels of ADCC activity, suggesting that Fc-mediated effector functions may contribute to protection through additional mechanisms (Figure 8D).

## Discussion

The purpose of this study was to construct NW alphavirus pseudoviruses based on the VSV system and establish a relatively safe and efficient DNA vaccine evaluation system suitable for a BSL-2 facility. In the study of NW alphavirus infection mechanisms *in vivo*, it was found that subcutaneous exposure more closely mimics natural infection via mosquito vector-borne transmission or by an accidental needle stick, while aerosol / intranasal exposure can be used to investigate the mechanisms of neural invasion[43]. Previous studies have demonstrated that in mouse models of NW alphavirus infection have demonstrated that EEEV can be detected at high titres in the blood, brain, spleen, lungs, and mesenteric lymph nodes of mice, with relatively lower levels in the liver and kidneys [44]. In mice infected with WEEV, viral loads are typically higher in the central nervous system (CNS), spleen, lungs, and peripheral lymph nodes [45]. VEEV infection leads to high viral titres in the brain, lymph nodes, spleen, liver, thymus, and other organs [46]. Consistent with these findings, our results demonstrated that NW alphavirus pseudoviruses were capable of infecting BALB/c mice via intraperitoneal, intravenous, and intracranial routes. However, no infection was observed via intranasal or subcutaneous routes. Following administration of pseudoviruses based on the highly virulent EEEV-V105 strain, bioluminescence signals were mainly detected in the brain, intestines, thymus, spleen, liver, lungs, and kidneys, which is consistent with prior reports [44]. Interestingly, bioluminescence signals in the kidneys were not observed in the EEEV-PE6 group, and the EEEV-SA group showed no detectable signals in either the liver or kidneys. For WEEV pseudovirus, bioluminescence signals were primarily observed in the brain, intestines, thymus, and spleen, which differs slightly from previous literature [45]. VEEV pseudovirus administration resulted in detectable signals detection in the brain, intestines, thymus, spleen, and liver, which was consistent with the literature [46]. These differences of tissue tropism, associated with various routes of pseudovirus administration, may be attributed to factors such as initial virus distribution, local tissue receptor expression, tissue barrier permeability, and immune clearance capacity.

Our findings suggest that NW alphavirus pseudoviruses, while capable of initiating infection through direct intracranial exposure or the circulatory system, likely lack the ability to cross the blood–brain barrier during a single round of infection.

It is important to note that Sindbis virus (SINV)-based chimeric systems have been developed for NW alphaviruses in recent years. These systems, which use a replication-competent alphavirus backbone bearing structural proteins of NW alphaviruses, have provided valuable insights into studies on viral biology, vaccine development, and antibody evaluation [35,47–49]. However, our use of the VSV-based pseudovirus system was motivated by its experimental flexibility and high packaging efficiency.

In addition to the SINV/VEE chimeric system, the virus-like replicon particle (VRP) system of NW alphaviruses has been widely used to produce pseudoviruses [50,51]. Compared with the VSV pseudovirus system, VRP system requires the co-transfection of two to three plasmids, including the replicon vector and helper RNAs, to generate particles that structurally resemble authentic alphaviruses. By contrast, the VSV pseudovirus system is structurally simple and operationally convenient, allowing flexible incorporation of heterologous viral envelope proteins.

In our vaccine evaluation experiments, DNA-immunized mice demonstrated enhanced protection against challenge with homologous strains, in line with the NAb data. Notably, immunization with the EEEV-V105 resulted in cross-neutralization of heterologous pseudovirus strains EEEV-PE6 and EEEV-SA, while EEEV-PE6 immunization significantly neutralized EEEV-V105 pseudovirus. Additionally, we established a correlation between NAb levels and *in vivo* protection, thereby enhancing the reliability of our vaccine evaluation methods.

As reported in previous studies, the replicon particle vaccines for EEEV, WEEV, or VEEV have elicited neutralizing antibodies with varying titres in mice [51]. In our study, The DNA vaccines for EEEV, WEEV, and VEEV induced different NAb levels. Given that the majority of VEEV neutralizing antibody epitopes are located on the E2 protein, further optimization of the E2 coding sequence may be necessary to enhance its expression and improve immunogenicity. Although not included in this study, analyzing serum binding antibodies targeting individual E1, E2, and E3 glycoproteins would help to further clarify the respective contributions of these antigen-specific responses to vaccine-induced protection.

In the VEEV DNA vaccine group, relatively low NAb titres were detected, and no strong linear correlation was observed between NAb levels and protection in mice. Interestingly, ADCC reporter assays revealed robust Fc-mediated effector activity in the sera. These findings imply that Fc-dependent mechanisms, such as ADCC, may contribute to protective immunity, highlighting the potential role of non-neutralizing functional antibodies in VEEV-induced protection. Recent studies have demonstrated that the NW alphavirus vaccines elicit protective immunity through both humoral and cellular immune responses [52–54]. Although the pseudovirus system employed in this study cannot evaluate cellular immune responses (e.g. ELISpot), future studies may incorporate live virus challenge models to assess cellular immunity more comprehensively.

The pseudovirus system developed here has significant value for the establishment of *in vitro* and *in vivo* infection models and the detection of NAb levels under BSL-2 conditions. However, it cannot recapitulate the complete viral life cycle, tissue tropism, or host–pathogen interactions observed with live viruses. Therefore, it is most appropriately applied for preliminary screening of vaccine candidates and antiviral drugs. Nevertheless, comprehensive validation of vaccine efficacy and immune correlates will require follow-up studies involving live virus challenges.

## Materials and methods

### Plasmids and cell lines

#### Plasmids

The codon-optimized E3-E2-E1 sequences of NW alphaviruses have been submitted to NCBI. The accession numbers are as follows: EEEV V105 (PV820777), EEEV PE6 (PV820776), EEEV-SA BeAr436087 (PV820778), WEEV EQ237_2024 (PV820780), VEEV TC-83 (PV820779). The coding sequences of NW alphaviruses were synthesized and cloned into the pcDNA3.1 vector, respectively forming the pcDNA3.1-EEEV-V105, pcDNA3.1-EEEV-PE6, pcDNA3.1-EEEV-SA, pcDNA3.1-WEEV, and pcDNA3.1-VEEV plasmids, which were used for pseudovirus construction and DNA vaccines. The glycoproteins are conjugated with an HA peptide tag at the amino terminus.

#### Cell lines

The cell lines used in this study included HEK293T (ATCC, CRL-3216), HEK293FT (Invitrogen, Carlsbad, CA, USA, R70007), HepG2 (ATCC, HB-8065), Vero (ATCC, CCL81), A549 (ATCC, CCL-185), HeLa (ATCC, CCL-2), BHK21(ATCC, CCL-10), K562 (ATCC, CCL-243), SK-N-MC (ATCC, HTB-10), CHO (ATCC, CRL-3440), Hep2 (ATCC,CCL-23), Huh7 (Japanese Collection of Research Bioresources [JCRB], 0403). SK-N-SH (ATCC, HTB-11), murine FcγRIII ADCC effector cells were obtained from Promega (USA, Cat. G7102).

### Phylogenetic analysis

The E3, E2, and E1 protein sequences of CHIKV (QKY67868.1), GETV (ABK32032.1), MAYV (QED21311.1), ONNV (AAC97205.1), RRV (AAA47404.1), SFV (NP_463458.1), SINV (AAM10630.1), EEEV V105 (PV820777), EEEV PE6 (PV820776), EEEV-SA BeAr436087 (PV820778), WEEV EQ237_2024 (PV820780), VEEV TC-83 (PV820779) were retrieved from NCBI. The unrooted phylogenetic tree was constructed using the maximum likelihood (ML) method in MEGA11 and evaluated by bootstrap resampling with 500 replicates.

### Pseudoviruses packaging using the VSV system

Taking EEEV-V105 as an example, pcDNA3.1-EEEV-V105 plasmid was used to transfect 293 T cells using Lipofectamine 3000 (Invitrogen, L3000015). Subsequently, G*ΔG-VSV-Fluc (VSV G pseudotyped virus) was diluted to 7.0×10^4^ TCID50/mL and then added to the cell culture flask. After 4–6 h, the culture medium was replaced with fresh DMEM medium, and the cells were cultured for an additional 24 h. The resulting cell culture supernatant was collected and filtered through a 0.45 µm pore-size membrane, yielding the pseudovirus suspension. This suspension was then aliquoted and stored at −80℃[55].

To package the prVSV-EEEV-V105-GFP pseudoviruses, the key differences in the packaging process were as follows: after transfection with the plasmid for 4–6 h, the transfected cells were then infected with G*ΔG-VSV, and the pseudovirus incubation period was 1–2 h[36, 56].

For pseudovirus titre determination, 4 × 10^4^ BHK21 cells/well were added and incubated for 24 h. The determination and calculation methods for prVSV-GFP-NW alphavirus pseudoviruses were as follows: the fluorescent spot counter (Cytation 5, Biotek) was used to quantify the number of GFP-positive cells, which was used to calculate the NW alphavirus pseudoviruses titres (focus-forming units per mL; FFU/mL)[57,58]. For prVSV-Fluc-NW alphavirus pseudoviruses, the positive well was determined as ten-fold relative luminescence unit (RLU) values higher than the cell background. The 50% tissue culture infectious dose (TCID50) was calculated using the Reed-Muench method [55].

### DNA vaccines and determination of vaccine efficacy

The study was approved (No. NIFDC (F) 2025(B)003) by the Animal Care and Use Committee at the National Institute for Food and Drug Control (NIFDC). Female 6-week-old BALB/c mice from the Institute for Laboratory Animal Resources, NIFDC were used in the study. The experimental group was immunized with the DNA plasmid at a dose of 100 µg per mouse, with a total of 7–8 BALB/c animals per group, and followed by electroporation. The control mice were injected with phosphate-buffered saline. Mice in experimental groups received DNA plasmid vaccinations at 0 w, 2 w, 4 w, and 7 w. Blood samples were collected from the cheek at 0 w, 3 w, 5 w, and 9 w. To verify the efficacy of the DNA vaccine against the viral strains, each mouse was intraperitoneally challenged with the prVSV-EEEV-SA-Fluc, prVSV-EEEV-PE6-Fluc, prVSV-EEEV-V105-Fluc, prVSV-WEEV-Fluc, and prVSV-VEEV-Fluc pseudovirus at a dose of 3.2×10^5^ TCID50, and bioluminescence data were collected 6 h later as described below.

### Bioluminescence imaging analysis

Mice received an intraperitoneal injection of the substrate, D-luciferin (50 mg/kg body weight), and were subsequently anesthetized with isoflurane 10 min after injection. Bioluminescent imaging (BLI) was monitored using the IVIS Lumina Series III Imaging System (PerkinElmer, Baltimore, MD, USA). The luciferase activity resulting from the pseudovirus infection in the body of the mice was detected using the Living Image software with a 60 s exposure. The bioluminescence signals from different regions of interest (ROIs) in the mice were measured using the photon-per-second mode with normalization for the imaging area (photons/s/cm^2^/sr; total lux).

### Animal model of infection

6-week-old, female BALB/c mice (2–4 mice per group) were used in the experiments. NW alphavirus pseudoviruses (prVSV-EEEV-SA-Fluc, prVSV-EEEV-PE6-Fluc, prVSV-EEEV-V105-Fluc, prVSV-WEEV-Fluc, prVSV-VEEV-Fluc) were used to infect the mice via the intracranial (ic) (1.6×10^5^ TCID50/mouse), intravenous (iv) (3.2×10^5^ TCID50/mouse), or intraperitoneal (ip) (3.2×10^5^ TCID50/mouse) route. The negative control group refers to the injection of mice with the prVSV-pcDNA3.1 pseudovirus. Imaging was performed 6 h post-injection. The experiment was validated through three independent replicate trials.

### Neutralization of pseudotyped viruses

The methods were adapted from our previous reports [49]. The prVSV-EEEV-V105-GFP, prVSV-EEEV-SA-GFP, prVSV-EEEV-PE6-GFP, prVSV-WEEV-GFP, and prVSV-VEEV-GFP pseudovirus titres were adjusted to between 1.1 × 10^7^ and 5.8 × 10^7^ FFU/mL. Then, the pseudovirus was mixed with serially diluted serum, incubated at 37°C for 1 h, and added to BHK21 cells for further incubation for 24 h. The number of AmCyan-positive cells is counted using Biotek. The 50% of neutralizing titre (NT_50_), which is calculated using the Reed-Muench method, serves as an indicator of the neutralizing antibody level.

### Western blot analysis

The 293 T cells were seeded into six-well plates. After 24 h, when the cell density reached approximately 70% confluence, the cells were transfected with 3 μg/well of the pcDNA3.1-EEEV-V105, pcDNA3.1-EEEV-PE6, pcDNA3.1-EEEV-SA, pcDNA3.1-WEEV, pcDNA3.1-VEEV, and pcDNA3.1-VSV G plasmids, respectively. After 24 h, the cells were collected and total protein was extracted using RIPA buffer. Protein expression was detected using a 4–20% acrylamide gradient SDS-PAGE gel. On the same membrane, E3E2E1 protein expression was detected using an HA antibody (Invitrogen, cat. 26183), while GAPDH protein expression, detected using a GAPDH antibody (Cell Signaling Technology, cat. 2118), was used to assess the sample loading amount. The pseudovirus supernatants were concentrated by ultracentrifuged at 30,000 rpm for 4 h. The viral particles were then resuspended in 200 µL of PBS and analyzed by western blotting. The expression of the E3E2E1 protein was detected using an HA antibody, while a VSV M protein antibody (Kerafast, cat. EB0011) was used as an internal control.

### Antibody-dependent cell-mediated cytotoxicity (ADCC) assay

An ADCC reporter bioassay core kit (Cat. G7102, Promega) was used to measure the induction of ADCC by no-NAbs in the serum of NW alphavirus DNA-immunized groups. Taking the VEEV group as an example, HEK293 T cells were seeded in 96-well plates at a density of 2.5 × 10^4^ cells/well and transfected with 200 ng of pcDNA3.1-VEEV for 24 h. After extensive washing, the cells were cultured in the presence or absence of the serum. The serums were serially diluted 1:3 in RPMI 1640 media (with 0.1% low-IgG FBS) from a starting dilution of 1:15 to a final concentration of 1:98415. Subsequently, 7.5 × 10^4^ effector cells that stably expressed FcγRIIIa and an NFAT response element driving the expression of firefly luciferase were added to each well. Luciferase activity was measured 6 h later according to the manufacturer's instructions (Promega). The data from each plate were adjusted by subtracting the average RLU value + 3 × SD from the blank wells, and the area under the curve (AUC) was calculated.

### Statistical analysis

All data analysis and graphing were performed using GraphPad Prism 8.0 software (GraphPad, San Diego, CA, USA). Data were analyzed using nonparametric one-way ANOVA and the Student *t*-test. The results are expressed as means ± SD. Statistical significance was indicated with asterisks (*P < 0.05, **P < 0.01, ***P < 0.001, and ****P < 0.0001).
